# Real-World Therapy Management and Outcomes of First-Line Axitinib Plus Pembrolizumab in Patients With Advanced Renal Cell Carcinoma in the United States

**DOI:** 10.3389/fonc.2022.861189

**Published:** 2022-05-19

**Authors:** Yousef Zakharia, Despina Thomaidou, Benjamin Li, Gordon Siu, Rebecca Levin, Anna Vlahiotis, Dharanija Rao, Giovanni Zanotti

**Affiliations:** ^1^ Holden Comprehensive Cancer Center, University of Iowa Hospitals and Clinics, Iowa City, IA, United States; ^2^ Pfizer Inc., Hellas, Greece; ^3^ Pfizer Inc., New York, NY, United States

**Keywords:** axitinib, combination, renal cell carcinoma, real-world, therapy management

## Abstract

**Background:**

Combination axitinib plus pembrolizumab is a standard of care in the first-line treatment of patients with advanced clear cell renal cell carcinoma (RCC). This analysis describes the clinical characteristics, treatment management and outcomes of patients receiving first-line (1L) axitinib plus pembrolizumab in a real-world US setting.

**Methods:**

Electronic health record (EHR)-derived data from the Flatiron Health Database, which includes ~280 cancer clinics across 800 sites in the US, were used. Patients had confirmed Stage IV or metastatic RCC and initiated 1L axitinib plus pembrolizumab on or after 1/1/2018 to 3/31/2021. Outcomes were best overall response rate; real-world progression-free survival (rwPFS) and overall survival (OS) at landmark time periods (3, 6, 9, and 12 months). Therapy management (TM) included dose hold, dose change and discontinuation. Data are reported as medians (IQR) unless otherwise noted.

**Results:**

355 patients received 1L axitinib plus pembrolizumab, with median follow-up of 9.7 (0.1–24.3) months. IMDC Risk Score was favorable, intermediate, and poor in 27 (7.6%), 126 (35.5%), and 76 (21.4%) patients, respectively (23.4% intermediate/poor, 12.1% unknown). 270 patients (76.1%) received only 1L axitinib plus pembrolizumab and 85 patients (24.3%) received ≥1 subsequent line of treatment; cabozantinib was the most frequent subsequent line of treatment (47.9%). rwPFS at 3 months and 1 year was 77.2% and 39.3%, respectively. OS ranged from 90.8% at 3 months to 73.5% at 1 year. Best overall response rate was 47.9%. Toxicity was the most common reason for first TM events of dose hold, change and discontinuation at, 58.6%, 58.5%, and 45.8%, respectively. Over 80% of patients with TM were able to continue with 1L axitinib plus pembrolizumab.

**Conclusions:**

In a real-world setting, axitinib plus pembrolizumab was effective as a 1L treatment for patients with advanced RCC. Dose holds, changes and discontinuation were driven by treatment-related toxicity. Dose holds may represent an effective TM strategy to toxicity.

## Introduction

In 2020, there were over 430,000 new cases of kidney cancer and approximately 180,000 associated deaths (1). Renal cell carcinoma (RCC) represents nearly 90% of kidney cancers and an estimated 75% of RCC cases of the clear cell histological subtype (2, 3). The 5-year relative survival rate for patients with localized kidney cancer is almost 93%; however for patients with advanced or metastatic disease, there is a dramatic fall to 14% (4).

The treatment of advanced RCC has recently taken substantial steps forward and continues to rapidly evolve. Existing and emerging first-line regimens include anti-angiogenic and/or immunosuppressive agents. These include vascular endothelial growth factor-receptor (VEGF-R) tyrosine kinase inhibitors (TKI; axitinib, cabozantinib, lenvatinib), and immune checkpoint inhibitors (avelumab, ipilimumab, nivolumab, and pembrolizumab). Treatment selection is partly based on the presence of risk factors as defined by prognostic models, predominantly the International Metastatic Renal Cell Carcinoma Database Consortium (IMDC) model and the Memorial Sloan Kettering Cancer Center model (5, 6). These models classify patients as favorable, intermediate or poor risk according to the number of risk factors. Combination treatment standards of care (preferred regimens) for patients of any IMDC risk include the combinations axitinib plus pembrolizumab (KEYNOTE-426) (7, 8), cabozantinib plus nivolumab (CheckMate 9ER) (9), and lenvatinib plus pembrolizumab (CLEAR trial) (10). In addition to the aforementioned combinations, nivolumab plus ipilimumab (CheckMate 214) (11, 12) is a preferred first-line treatment option for patients of IMDC intermediate or poor risk (13–15).

The axitinib plus pembrolizumab combination was approved for the first-line treatment of advanced RCC by the FDA in April, 2019, based on the results of the KEYNOTE-426 phase 3 trial (7, 8). In an extended follow-up of the phase 3 trial, axitinib plus pembrolizumab showed sustained clinical benefit compared with single agent sunitinib in both overall survival (OS; median not reached with pembrolizumab and axitinib vs. 35·7 months [95% CI 33·3–not reached] with sunitinib) and median progression-free survival (PFS; 15·4 months [12·7–18·9] vs. 11·1 months [9·1–12·5]; p<0·0001) (7).

In the KEYNOTE-426 phase 3 extended follow-up, treatment-related adverse events led to approximately one fifth of patients discontinuing axitinib treatment and nearly two thirds requiring treatment interruption (7). Understanding the clinical characteristics of patients with advanced RCC treated with axitinib plus pembrolizumab may help identify populations and strategies to optimize treatment duration and potentially improve clinical outcomes. This analysis aimed to describe the demographic and clinical characteristics, treatment patterns, and therapy management of patients with advanced RCC treated with first-line combination axitinib plus pembrolizumab in a US real-world setting.

## Methods

### Data Source and Patients

EHR-derived data from the nationwide Flatiron Health de-identified database, which includes approximately 280 cancer clinics across ~800 sites of care in the US. Eligible patients had an RCC diagnosis (ICD9 189.x or ICD10 C64x or C654x), with evidence of stage IV or recurrent metastatic RCC with a metastatic diagnosis date on or after January 1, 2011. Patients were included if they were 18 years or older in the year of the index first line therapy prescription, had evidence of pathology consistent with RCC, and had at ≥2 clinic encounters on different days to be included in the study. Among these, 355 patients initiating first-line axitinib + pembrolizumab on or after 1/1/2018 to 3/31/2021, with no prior aRCC treatment were identified for the study. First line therapy cohorts were explored regardless of follow-up time available, except for real-world PFS (rwPFS) and real-world OS (OS) where patients were censored (see below). Baseline characteristics are considered as of the index date, which was defined as the date of first prescription of first-line therapy. This study used only de-identified EHR-derived data. The protocol from Flatiron Health governing data collection had IRB approval with a waiver of informed consent, in accordance with the Health Insurance Portability and Accountability Act.

### Derived Variables and Outcomes

IMDC Risk Score was derived by Flatiron Health from individual data components as available in the EHR. When missing data did not allow patients to confidently be grouped into ‘Favorable’, ‘Intermediate’, or ‘Poor’ risk status according to the validated algorithm, Flatiron Health used the additional classifications of ‘Poor/Intermediate’ and ‘Unknown’ Risk.

Patient follow-up was defined as time from index date to last recorded activity. Treatment duration was calculated as the time from first treatment date to last treatment date, regardless of any gaps in treatment. Time to treatment failure (TTF) was defined as the time from treatment initiation until treatment discontinuation or therapy change (next line of therapy – switch or augmentation), end of enrolment, or death. Patients were censored in the analysis of rwPFS and OS if they did not experience a respective clinical event (rwPFS: progression/death; OS: death) as of the last confirmed structured or unstructured activity date, and were still alive at the study cut-off date.

Real-world response was defined using response assessment categories that are abstracted based on the healthcare provider’s qualitative description of response to therapy. Best overall response was defined as the maximum response to therapy to first-line therapy (complete response plus partial response). Therapy management events, as documented in the EHR, were defined as dose hold, dose change and discontinuation. For patients with more than one reason for therapy management, a hierarchy was used to select the reason: toxic effect of treatment > progression > cancer-related symptoms not due to treatment > non-cancer related medical issue > financial > patient request > no evidence of disease > insufficient response > other > unknown. Subsequent clinical events such as treatment switches after toxicity-related therapy management were also captured.

### Statistical Analysis

Descriptive statistics were used to summarize patient demographic and clinical characteristics, treatment patterns and therapy management, and to tabulate landmark rwPFS TTF and OS. The Kaplan-Meier method was used to estimate rwPFS, rwTFF, and OS curves.

## Results

### Patients

Three hundred and fifty-five patients received first-line axitinib plus pembrolizumab, with a median (IQR) follow-up of 9.67 (4.37-14.83) months. Patient demographics and clinical characteristics are summarized in **Table 1**. Overall, the majority of patients were of white ethnicity (67.89%), male (69.58%), and median age was 68.00 (60.00-75.00) years. Patients were predominantly treated at community-based practices (n=330, 92.96%), with 25 patients (7.04%) treated at academic-based practices.

**Table 1:** Patient demographics and clinical characteristics.

	Axitinib plus pembrolizumab
	N = 355
Follow-up, median (IQR), months	9.67 (4.37, 14.83)
Age, median (IQR), years	68.00 (60.00, 75.00)
Gender, n (%)	
Female	108 (30.42)
Male	247 (69.58)
Race, n (%)	
Asian	3 (0.85)
Black	20 (5.63)
White	241 (67.89)
Other	53 (14.93)
Missing	38 (10.70)
Stage at diagnosis, n (%)	
I – III	151 (42.53)
IV	197 (55.49)
Missing	7 (1.97)
Histology, n (%)	
Chromophobe	3 (0.85)
Clear cell	274 (77.18)
Papillary	15 (4.23)
RCC, NOS	58 (16.34)
Translocation	2 (0.56)
Other	3 (0.85)
Nephrectomy, n (%)	
Yes	197 (55.49)
No	158 (44.51)
ECOG performance score, n (%)	
0	130 (36.62)
1	112 (31.55)
2	39 (10.99)
3	12 (3.38)
Missing	62 (17.46)
IMDC risk score, n (%)	
Favorable	27 (7.61)
Intermediate	126 (35.49)
Poor	76 (21.41)
Poor/Intermediate*	83 (23.38)
Unknown	43 (12.11)

*Patients had an IMDC score of 1–2 with missing data for ≥1 of the other IMDC risk factors, and classification into Intermediate or Poor, separately, could not be made using available data.

ECOG, Eastern Cooperative Oncology Group; IMDC, International mRCC Database Consortium; NOS, not otherwise specified; RCC, renal cell carcinoma.

At diagnosis, 55.49% of patients had Stage IV, 42.54% had Stage I - III RCC (1.97% Stage at diagnosis unknown), and 77.18% of patients had clear cell histology. More than half of patients had undergone a nephrectomy, almost 70% of patients had a ECOG performance score of 0 or 1, and the largest prognostic group was intermediate IMDC risk (35.5%; **Table 1**).

### Treatment Characteristics

At the time of analysis, 270 patients (76.06%) had initiated first-line therapy with axitinib plus pembrolizumab (**Table 2**) and had not received subsequent therapy after first-line treatment. Over 50% of all patients had discontinued axitinib plus pembrolizumab treatment at the time of analysis, whereas 20% continued with axitinib plus pembrolizumab treatment (**Table 2**). The majority of patients received axitinib plus pembrolizumab according to the recommended dose (axitinib 5mg; 93.8%) and dose schedule (twice daily; 96.3%) (**Table 2**). Median treatment duration was 163 (IQR 69–335) days (censored and uncensored). After controlling for consistent follow-up of 180 days, median treatment duration for 239 patients was 269 (IQR 150-390) (**Table 2**).

**Table 2:** Treatment characteristics.

	Axitinib plus pembrolizumab
	N = 355
Duration of treatment, median (IQR), days	163 (69, 335)
Initial axitinib dose, n (%), mg	
3	14 (3.94)
5	333 (93.80)
7	1 (0.28)
10	3 (0.85)
Other	4 (1.13)
Initial dose schedule, n (%)	
Once daily	7 (1.97)
Twice daily	342 (96.34)
Other/Unknown	6 (1.69)
Treatment pattern*	
Axitinib plus pembrolizumab	270 (76.06)
Axitinib plus pembrolizumab → cabozantinib	31 (8.73)
Axitinib plus pembrolizumab → ipilimumab, nivolumab	7 (1.97)
Axitinib plus pembrolizumab → everolimus, lenvatinib	4 (1.13)
Axitinib plus pembrolizumab → pazopanib	4 (1.13)
Other	39 (10.98)
Duration of treatment for patients with ≥180 days follow-up, median (IQR), days	260 (150, 390)
Pembrolizumab plus axitinib treatment status at the time of analysis, n (%)	
Augmentation	2 (0.56)
Continuation	71 (20.00)
Discontinuation	199 (56.06)
Switch	83 (23.38)

*Treatment patterns as reported for >1% of patients. Treatment patterns include patients who were censored (still on treatment, and those who died), as well as those with short follow-up.

Eighty-five patients (23.9%) received more than one line of therapy, with VEGF-R inhibitors as the most common second-line treatment (51/85 patients, 60.0% in **Table 2**). Cabozantinib was the most frequently used second-line agent (43/85 patients, 50.1%), followed by nivolumab plus ipilimumab (9/85 patients, 10.6% in **Table 2**).

### Clinical Events and Treatment Effectiveness

**Figure 1:**
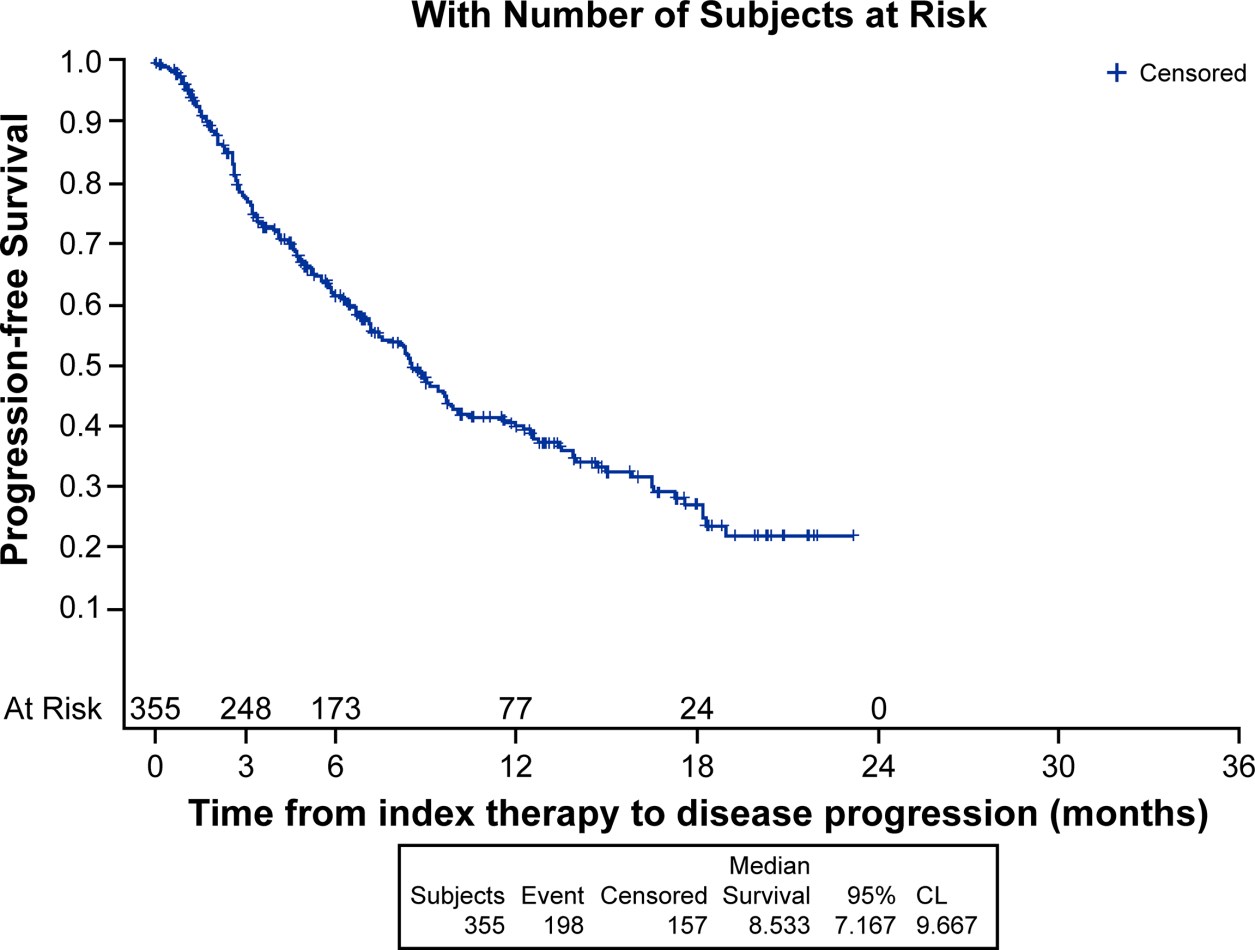
Kaplan-Meier analysis of real-world progression free-survival.

**Figure 2:**
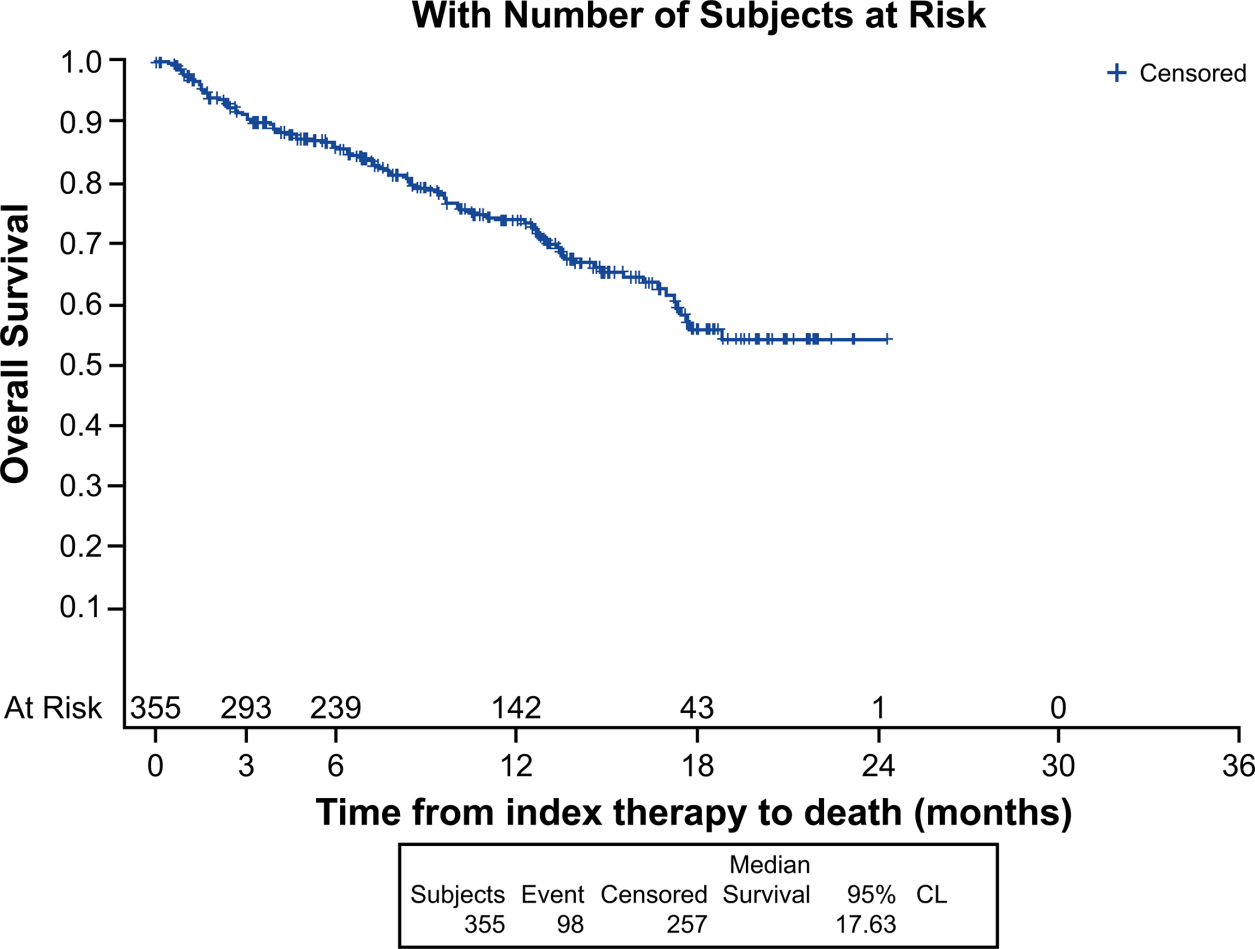
Kaplan-Meier analysis of real-world overall survival.

**Figure 3:**
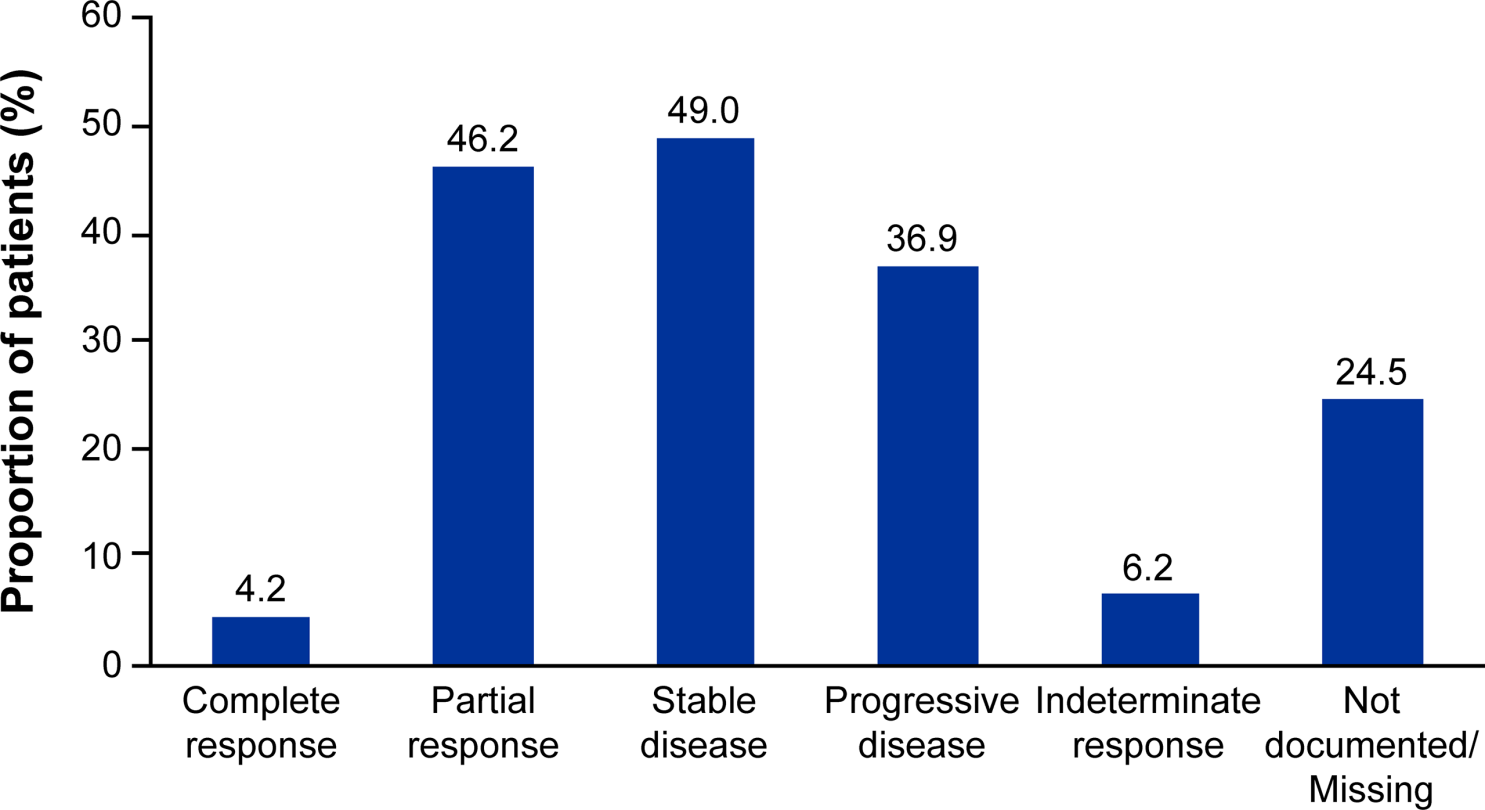
Patient response to first-line axitinib plus pembrolizumab treatment. Responses are not mutually exclusive and represent patient responses throughout the entire follow-up period.

Median (95% CI) Kaplan-Meier estimates of TTF (17.75% censored) [figure not shown] and rwPFS (44% censored) were 2.70 (2.20, 3.00) and 8.53 (7.17, 9.67) (**Figure 1**) months, respectively. Median OS (257 censored) was not reached (**Figure 2**). **Table 3** describes TTF, rwPFS, and OS during follow-up at specified landmark timepoints (3, 6, 9, and 12 months). Patient response to treatment is summarized in **Figure 3**. The best overall response rate to axitinib plus pembrolizumab was 47.9% (170/355 patients); complete response was observed in 4.23% of patients and partial response observed in 46.20%.

**Table 3:** Patients experiencing clinical events during follow-up at specified landmark timepoints.

	Axitinib plus pembrolizumab, N = 355		
	Time to treatment failure, n (%)	Real-world PFS, n (%)	Real-world OS, n (%)
≥3 months	147 (41.41)	247 (77.22)	291 (90.75)
≥6 months	71 (20.00)	172 (61.19)	237 (85.70)
≥9 months	33 (9.30)	110 (46.96)	186 (78.89)
≥12 months	22 (6.20)	75 (39.30)	138 (73.54)

OS, overall survival; PFS, progression-free survival.

### Therapy Management and Subsequent Events

**Figure 4:**
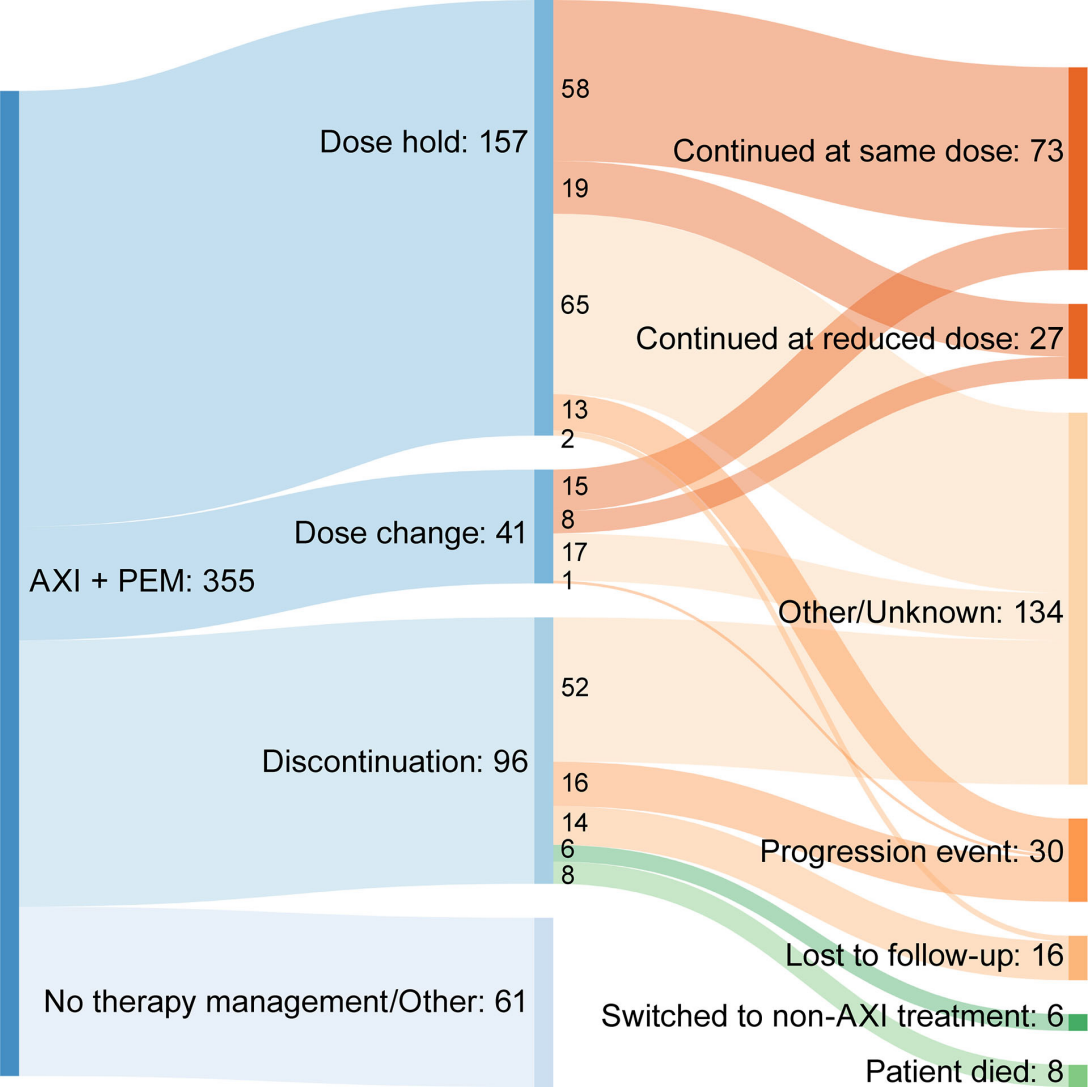
Sankey diagram summarizing subsequent events following therapy management due to toxicity. AXI + PEM, axitinib plus pembrolizumab.

Median duration of treatment for patients without treatment modifications was nominally shorter compared to patients who received treatment modifications at 122 and 176 days, respectively. The most frequent first therapy management was dose hold (157 patients, 44.23%), followed by discontinuation (96 patients, 27.04%) and then dose change (41 patients, 11.55%). For each type of therapy management, toxicity related to treatment was the most common reason (**Table 4**). Time to first therapy management was similar for dose hold (42 days) and dose change (42 days), but nominally longer for discontinuation (71 days). Subsequent events following therapy management due to toxicity of therapy are summarized in **Figure 4**. The majority of patients who had a dose hold or change as their first therapy management (due to toxicity of therapy) continued treatment with axitinib plus pembrolizumab at their current dose, or at a reduced dose after therapy management (**Table 4**). For patients whose first therapy management was discontinuation due to toxicity of therapy, 13.6% (6/44) switched to another treatment (**Table 4**).

**Table 4:** First therapy management and subsequent events after therapy management due to toxicity.

	Patients with Therapy Management, n = 294		
	Dose hold	Dose change	Discontinuation
**First event for patients with therapy management, n (%)**	**157 (44.23)**	**41 (11.5)**	**96 (27.04)**
Time to first therapy management, median (IQR), days	42 (20, 93)	42 (21, 71)	71 (31.5, 139.5)
Reason for first therapy management, n (%)			
Treatment-related toxicity	92 (58.60)	24 (58.54)	44 (45.83)
Progression	0	1 (2.44)	32 (33.33)
Cancer-related symptoms not associated with treatment	7 (4.46)	0	4 (4.17)
Non-cancer medical issue	35 (22.9)	0	6 (6.25)
Financial	1 (0.64)	0	3 (3.13)
Patient request	2 (1.27)	0	2 (2.08)
Other/Unknown	20 (12.73)	16 (66.67)	5 (5.21)
Subsequent event after therapy management due to toxicity, n (%)			
Continued axitinib treatment at same dose	58 (63.04)	15 (62.50)	–
Continued axitinib at reduced dose	19 (20.65)	8 (33.33)	–
Switched to non-axitinib treatment	0	0	6 (13.64)
Progression event	13 (14.13)	1 (4.17)	16 (36.36)
Death	0	0	8 (18.18)
Lost to follow-up	2 (2.17)	0	14 (31.82)

## Discussion

The introduction of combination anti-angiogenic agents with immunotherapy agents to treat patients with advanced RCC has provided substantial clinical benefit; however, this has also added to the complexity of managing treatment-related toxicity. For TKIs, such as axitinib, treatment-related toxicity may be managed by dose holds or reduction, whereas, for immunotherapy agents such as pembrolizumab, toxicity management may include the administration of immunosuppressants, such as corticosteroids (16). Some of the treatment-related adverse events associated with anti-angiogenic TKIs are similar to those seen with immunotherapy agents and may be additive as well as overlapping, which adds to the complexity of identifying the etiology and managing accordingly (16). In our analysis, the most common first therapy management for patients receiving axitinib plus pembrolizumab was dose hold (44.2%), followed by discontinuation (27.0%) and then dose change (11.5%). In each instance, the most frequent reason for therapy management was treatment-related toxicity. Although the etiology of the treatment-related toxicity was not recorded in our analysis, our data support the use of dose hold as an effective means of treatment management. Following dose hold, nearly 85% of patients with a dose hold due to toxicity of therapy continued their current axitinib dose, or continued axitinib combination therapy at a modified dose or schedule. Similarly, for patients whose first therapy management was dose modification (reduction), over 90% with a dose modification due to toxicity of therapy were able to continue receiving axitinib plus pembrolizumab. These results are of supportive of the implications of previous research suggesting that dose titration may be a potential means for improving outcomes (17).

Patients who discontinued treatment as a first therapy management had a median time to discontinuation of 71 days, representing approximately two cycles of axitinib treatment. Almost half of these discontinuations were due to treatment-related toxicity. This represents a sizable proportion of patients who discontinue before any other therapy management strategies are employed. Following axitinib plus pembrolizumab discontinuation due to toxicity, almost 14% switched to another treatment after discontinuing. These patients may represent a group who may benefit from optimized therapy management. As several different combinations of immunotherapy and TKIs are approved in metastatic RCC and other malignancies, it is critical for the prescribing providers to be comfortable managing any adverse events and be familiar with the strategies of holding and modifying the responsible agents according to the grade of toxicities and prior to premature discontinuation, which may negatively impact the clinical outcome.

To our knowledge, this analysis is the first-real-world data reporting patient characteristics, treatment management and outcomes in patients with advanced RCC who received first-line axitinib plus pembrolizumab. Randomized controlled trials (RCTs) generally utilize stringent eligibility criteria to ensure high internal validity to help answer a specific clinical question. As a result, the RCT population of patients do not always share the same diversity as those seen in a real-world clinical setting (18). In real-world practice, patients with advanced RCC treated with anti-angiogenic TKIs have been shown to be of less favorable prognosis, have more co-morbidities and worse outcomes compared with patients enrolled on RCTs (19, 20). Consistently, there were fewer patients from our analysis of predominantly community-based US clinical practices, who were of IMDC favorable risk (7.6%), compared with those who enrolled on the pivotal phase 3 trial (32%) (7, 8). Notably, 12.1% of patients in our analysis were of unknown IMDC risk score and 23.4% could not confidently be classed as only intermediate or poor risk due to missing data. This means that it is possible >50% of the analyzed population may have been of poor IMDC risk. Despite this, response to axitinib plus pembrolizumab in our analysis was of a similar magnitude compared with that reported in KEYNOTE-426 (ORR, 47.9% vs. 60%, respectively) (7). KEYNOTE-426 also noted the most common reason for discontinuation of therapy among all patients in the analysis was disease progression (7). This analysis, however, did not present the most common reason for discontinuation among all patients who discontinued; rather, but reported the most common reasons for discontinuation only when discontinuation was the first therapy management ‘event’ a patient experienced after initiation of first-line axitinib plus pembrolizumab. In a separate analysis that utilized the Flatiron database, patients with advanced RCC treated with first-line axitinib plus pembrolizumab had 12-month rwPFS and OS of 41.4% and 68.5%, respectively (21). These estimates are similar to the wPFS (39.3%) and OS (73.5%) at 12 months reported in our analysis.

The main limitation of this analysis was the short follow-up period of approximately 10 months. This resulted in a relatively short duration of treatment and the number of patients with ≥180 days follow-up was insufficient to allow for a meaningful analysis of therapy management events. This impacted the analysis of outcomes and treatment patterns. The inclusion of patients with short follow-up may have artificially decreased the proportion of first-line axitinib plus pembrolizumab patients who advanced to subsequent treatment. It should also be noted that for therapy management (dose hold, change, discontinuation), the denominator was patients who had a *therapy management event* attributable to toxicity of treatment, not patients who experienced toxicity of treatment. Specific adverse events, including those indicating toxicity of treatment, were not explicitly captured. Therefore, the category of patients who received therapy management may not represent all patients who experienced toxicity due to therapy. As with many real-world studies, missing data, or data elements not captured, may suggest that some conclusions drawn from this analysis need further investigation. Such examples relevant to this analysis are the lack of information from this RWD source about the absence or presence of sarcomatoid tumor features, or type and grade of toxicity leading to treatment discontinuation. Nevertheless, this analysis provides an important insight into the clinical characteristics and therapy management of patients with advanced RCC receiving axitinib plus pembrolizumab in a real-world setting. Furthermore, it identifies dose hold as a potentially effective treatment management strategy.

## Data Availability Statement

The raw data supporting the conclusions of this article will be made available by the authors, without undue reservation.

## Ethics Statement

The studies involving human participants were reviewed and approved by Flatiron Health governing data collection IRB with a waiver of informed consent, in accordance with the Health Insurance Portability and Accountability Act. This study used only de-identified EHR-derived data. Written informed consent for participation was not required for this study in accordance with the national legislation and the institutional requirements.

## Author Contributions

Conception and design: All authors. Performed research: All authors. Contributed new reagents or analytic tools: N/A. Analysis and interpretation of data: All authors. Drafting of the manuscript: All authors. Critical revision of the manuscript for important intellectual content: All authors. All authors contributed to the article and approved the submitted version.

## Conflict of Interest

The authors declare that this study received funding from Pfizer Inc. RL, BL, GS, GZ, DT, AV, and DR were employees of Pfizer Inc. The funder had the following involvement with the study: Pfizer-affiliated authors were involved in the study design, collection, analysis, interpretation of data, the writing of this article, and the decision to submit it for publication.

The remaining authors declare that the research was conducted in the absence of any commercial or financial relationships that could be construed as a potential conflict of interest.

## Publisher’s Note

All claims expressed in this article are solely those of the authors and do not necessarily represent those of their affiliated organizations, or those of the publisher, the editors and the reviewers. Any product that may be evaluated in this article, or claim that may be made by its manufacturer, is not guaranteed or endorsed by the publisher.
